# The Role of Canine Models of Human Cancer: Overcoming Drug Resistance Through a Transdisciplinary “One Health, One Medicine” Approach

**DOI:** 10.3390/cancers17122025

**Published:** 2025-06-17

**Authors:** Sara Gargiulo, Lidovina Vecchiarelli, Eleonora Pagni, Matteo Gramanzini

**Affiliations:** 1 Institute of Clinical Physiology, National Research Council, Via Fiorentina 1, 53100 Siena, Italy; 2 Core Research Laboratory (CRL), Istituto per lo Studio, la Prevenzione e la Rete Oncologica (ISPRO), 53100 Siena, Italy; 3 Animal Welfare at Animal and Plant Health Agency, Department for Environment Food and Rural Affairs, Stafford ST18 0AR, UK; 4 Veterinary Medical and Oncology Centre “Etruria”, Via Sandro Pertini 5-6, Monteriggioni, 53035 Siena, Italy; eleonora@clinicaveterinariaetruria.it; 5 FSA (Fondazione Salute Animale), Via Trecchi 20, 26100 Cremona, Italy; 6 Institute of Crystallography, National Research Council, Strada Provinciale 35d, n. 9-00010, Montelibretti, 34149 Rome, Italy; matteo.gramanzini@cnr.it

**Keywords:** drug resistance, comparative oncology, canine model, spontaneous tumor systems, “one health, one medicine”

## Abstract

Introduction: Chemotherapy is a primary treatment option in human and veterinary oncology. Like humans, canine patients often develop drug resistance. Comparative oncology is gaining increasing interest, and spontaneous tumors of companion dogs have emerged as a powerful resource for better understanding human cancer. The genetic, molecular, and histological features of tumors in dogs are more closely related to those in humans than the ones in laboratory animals, including complex mechanisms of drug resistance. Methods: A comprehensive literature search was conducted in the electronic database Clarivate Web of Science (WOS): Medical Literature Analysis and Retrieval System Online (MEDLINE) from 1990 to 2025 (updated 22 January 2025). The final set includes 59 relevant full-text English articles. Results: The literature findings suggest that canine spontaneous tumors are valuable model systems with important translational implications for identifying novel mechanisms of chemotherapy resistance shared with humans and may help advance the current standard of care in precision medicine. Conclusions: We have provided an updated overview of the role of canine tumor models to study oncotherapy resistance, focusing on limitations and opportunities for advancement. Despite complementary benefits of such models in translational oncology research, their relevance remains underestimated. Strengthening the collaboration between human and veterinary medicine professionals and comparative medicine researchers, and obtaining the support of interdisciplinary institutions, could contribute to addressing the problem of multidrug resistance for both human and canine patients. Future research may promote using canine spontaneous tumors as translational therapeutic models for human chemoresistance, through a multidisciplinary approach based on the emerging “One Health, One Medicine” paradigm.

## Simple Summary

Resistance to oncotherapy represents a significant challenge for both human and canine patients. Comparative oncology has highlighted remarkable similarities between canine and human tumors with respect to genetic and tissue composition factors involved in drug resistance. Spontaneous canine cancer models more accurately recapitulate the oncogenesis and complex, heterogeneous microenvironment of human tumors than laboratory animal models in which neoplasia is induced do. Companion dogs share numerous exposure factors with humans and show clear similarities in molecular and histological features, as well as in immune and treatment responses. An appropriate model is essential for successfully implementing precision medicine strategies. This comprehensive review aims to highlight the critical role of canine models of human cancer in advancing current knowledge of drug resistance mechanisms, helping to improve the development of treatments that address this relevant issue through a “One Health, One medicine” translational research approach.

## 1. Introduction

The identification of mechanisms underlying primary and secondary resistance to therapies in oncology is an active field of research.

Additionally, the need to promote an interdisciplinary approach in oncology and to improve the choice of valid experimental preclinical models to address the problem of drug resistance (DR) in cancer treatment is widely recognized [1].

Nowadays, canine cancer cell lines and spontaneous canine tumors have been recognized as valuable models for studying cancer biology and testing novel therapeutic approaches in vitro and in vivo that could benefit both species. However, efforts to integrate collaboration between human and veterinary medicine, both in clinical and research settings, are still under development, hindering the possibility of knowledge sharing and optimizing the expertise and data potentially available to develop more effective strategies to defeat DR [2].

Companion dogs can be affected by many of the same types of cancer that occur in humans, including breast, prostate, and lung cancers, glioma, melanoma, and lymphoma/leukemia; comparative oncology studies have highlighted extensive histological, genetic, and molecular similarities between the two species [3,4]. Furthermore, comparative cancer epidemiology through Animal Cancer Registries has been recognized as a precious source of complementary information for human oncological prevention [5]. However, the role of animal models of human cancer in clinical therapeutic research is still a matter of debate. Overall, tumor biology appears to be more closely related among spontaneous tumors occurring in both dogs and humans than those are induced in immunocompromised laboratory rodents by chemicals, human tumor cell transplantation, or genetic engineering techniques [3,6]. Dogs are exposed to similar environmental risk factors to humans compared to the controlled artificial parameters of a laboratory animal facility. Canine models may also better reflect the complexity of the tumor microenvironment and its interaction with host immunity, influencing the therapeutic response [7]. Furthermore, the breed-specific predisposition for certain types of tumors allows us to investigate the role of genetics in tumor development [8]. Also, the shorter life expectancy of dogs and their faster rate of cancer progression compared to humans reduce the length of clinical studies and the time needed to extrapolate useful results on DR to humans, at lower costs and with less stringent regulatory requirements [9,10]. Considering the current prevalence and social role of dogs in Western world families, and the modern ethical attitude aimed at protecting their wellbeing and health, sample collection, surgical interventions, and imaging are currently widely implemented in dogs with cancer. Indeed, clinical veterinary medicine has reached a high level of specialization, applying diagnostic and therapeutic standards in veterinary medical centers comparable to human medicine. In general, the rate of the effective translation of therapies from studies on rodent models to clinical trials is very low [11]. Interestingly, dogs are often treated with the same chemotherapeutic drugs used in human patients and, after initial success, develop similar DR. Therefore, clinical investigations on companion dogs could be well accepted by the public opinion, since they will bring benefits beyond humans to veterinary oncology.

The attractiveness of the canine comparative models in the fight against cancer of humans has been emerging since 2003, when the National Cancer Institute’s Center for Cancer Research (CCR) started the Comparative Oncology Program (NCI-COP; https://ccr.cancer.gov/comparative-oncology-program, accessed on 19 February 2025) [11]. Preclinical studies in dogs with cancer have helped to test innovative approaches of immunotherapy and gene therapy, as well as optimized drug delivery tools before human clinical trials [7]. Furthermore, canine cancer cell lines, both continuous and derived from primary tumors, have been advantageously used as an in vitro model to study the molecular pathways involved in tumor development and resistance to chemotherapeutics.

DR is a relevant clinical challenge limiting the success of chemotherapy in both human oncology and veterinary medicine, and the cellular mechanisms of DR in dogs appear to overlap with those in humans due to the evolutionarily conserved genetic pathways involved [12,13]. Despite the emerging promotion of the One Medicine paradigm and One Health education, these powerful animal models remain vastly underutilized to study cancer biology and DR, likely due to limited opportunities for interdisciplinary convergence between clinical and research institutions. Indeed, identifying and targeting DR tumor cells appears to be a rational approach to improve current standards of care, and dogs may represent a valuable large animal model to study this research challenge, thereby facilitating the use of approaches similarly to humans. Canine models could contribute to gain new insights into the development of anticancer drugs that promote the reversal or prevention of DR; their complementary use with other available experimental models would ensure more comprehensive results. Clearly, dogs and humans may show species-specific differences in the pharmacokinetic and pharmacodynamic profiles of drugs that could potentially affect their efficacy and safety [14]. An overview of current knowledge on chemotherapy resistance is beyond the scope of this review. Our aim is to explore the contribution of canine models in the study of tumor resistance to chemotherapy, the capabilities and shortcomings of research efforts, and possible strategies to effectively integrate available resources to address this specific public health challenge with a high social impact.

From this perspective, a constructive scientific debate could be stimulated among researchers and clinicians on the opportunity to implement an integrated use of animal models to provide new insights into overcoming resistance to chemotherapy drugs.

## 2. Materials and Methods

### 2.1. Search Strategy

The search strategy was designed to capture all relevant papers investigating the use of spontaneous canine tumors as models to study the DR issue. This research project was prospectively (pre)registered in Open Science Framework (OSF) (https://osf.io/h8veq/?view_only=d3b2241cacc64c87afa615cd1fa2ff6f, accessed on 5 February 2025; DOI 10.17605/OSF.IO/H8VEQ). The Clarivate Web of Science (WOS): Medical Literature Analysis and Retrieval System Online (MEDLINE) electronic database was searched and screened on 22 January 2025 using the following string of search terms: TS = ((canine model ) AND (human) AND (neoplasia OR tumor) AND (oncotherapy OR chemotherapeutic OR drug) AND (resistance)) to identify pertinent articles from 1990 to 2025 (updated 20 January 2025). The final set includes 89 full-text English articles, which were exported into a single Reference Manager file (Supplementary Materials, RM file).

### 2.2. Selection Criteria

For inclusion in this study, the following were considered eligible: peer-reviewed case reports, comparative studies, classical articles, journal articles, journal reviews, randomized controlled trials, Research Support, Non-U.S. Gov’t, Research Support, U.S. Gov’t, P.H.S, Research Support, N.I.H., Extramural, and technical reports that focused on spontaneous canine tumors models for the overall DR study. Titles, abstracts, and full texts were critically examined for relevance and the following articles were excluded:

- Articles referring to resistance to therapies other than anticancer drugs (e.g., antimicrobials, antiparasitics) and clearly outside the scope of the review;
- Articles where DR was not the primary focus of investigation (e.g., compound toxicity, targeting strategies, or drug delivery);
- Articles where the in vitro/in vivo canine model does not have an evident relevance in the context of the study;
- Articles in which animal models other than canine models were used (i.e., laboratory mice or other companion animals) and clearly outside the scope of this review;
- Reviews that summarize the results of relevant original articles already included in the search list;
- Articles in which the preliminary data are provided by the same authors of a main work.

**Figure 1:**
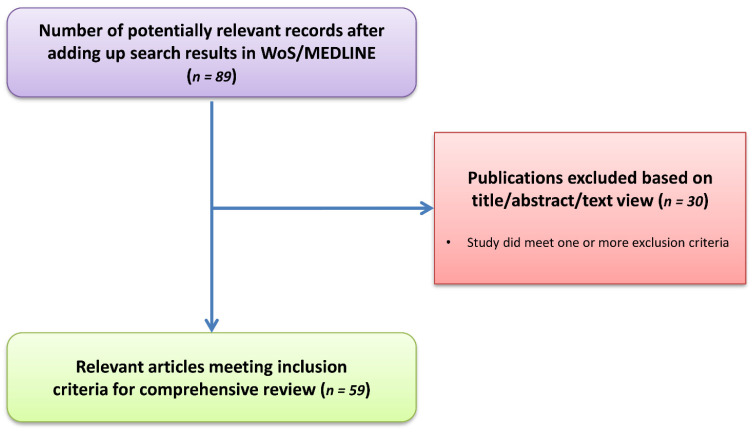
Flow diagram of the manuscript selection process.

Ultimately, 59 articles were identified as appropriate to summarize the findings of the present study, as presented in Figure 1.

## 3. Results

In this review, we discuss the literature findings that highlight the value of canine models in comparative oncology, based on the main mechanisms of DR shared with humans. Although we have selectively discussed the spectrum of the most relevant DR biological pathways displayed by tumor cells, the relationship between them is obviously more complex.

**Figure 2:**
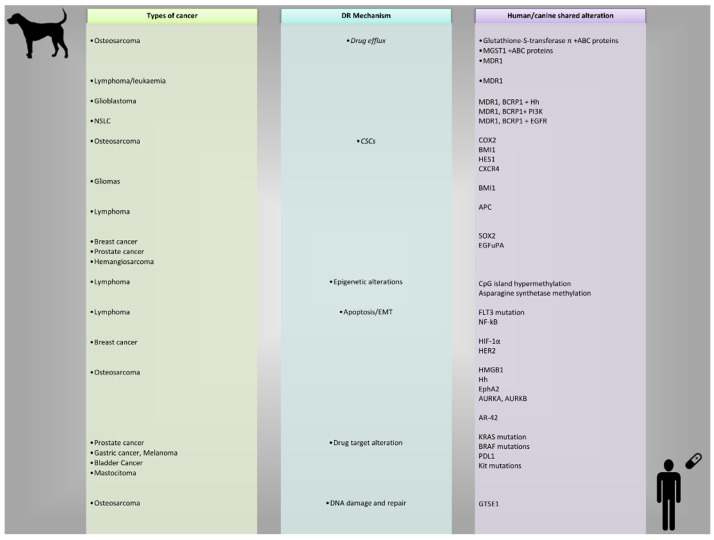
Schematic diagram outlining comparative oncology in companion dogs and humans.

A comparative summary of DR mechanisms shared by human and canine tumors is shown in Figure 2.

### 3.1. Efflux of Chemotherapeutic Agents

Enhanced drug efflux by the ATP-binding cassette (ABC) superfamily of transmembrane transport proteins is one of the most frequent mechanisms of DR. Their expression is often elevated in chemotherapy-resistant tumors, both in humans and dogs, resulting in active efflux of chemotherapeutic agents. In particular, the overexpression of the P-glycoprotein (P-gp), also known as multidrug resistance 1 (MDR1), multidrug resistance-associated protein 1 (MRP1), multidrug resistance-associated protein 2 (MRP2), and breast cancer resistance protein (BCRP), has been linked to multidrug resistance (MDR) and poor prognosis in both human and canine patients by hindering the intratumoral uptake of chemotherapeutics. Interestingly, ABC transporters are constitutively expressed by the blood–brain barrier (BBB), a further challenge for effective pharmacotherapy against brain tumors. Based on this evidence, the combined use of conventional drugs and ABC transporter inhibitors has been proposed to manage MDR.

Among the first animal models that highlighted the potential efficacy of P-gp blockers against MDR were Collie dogs that showed increased sensitivity to the neurotoxic effects of ivermectin, a widely used antiparasitic and P-gp substrate, which was related to an impaired P-gp function in the BBB due to a mutation in the MDR1 gene. Consistent effects of ivermectin were shown in studies on a mouse model lacking the P-gp function, which led to a compromised BBB and subsequent increased central nervous system (CNS) permeability to xenobiotics [15]. Overall, this dog breed represents a useful model of naturally occurring gene invalidation, which could be exploited to inform human therapies targeting MDR mechanisms.

Similarly, one of the first clinically relevant mechanisms of chemoresistance demonstrated was cisplatin resistance mediated by the enzyme glutathione-S-transferase π in a spontaneous model of canine osteosarcoma [16]. The validity of this spontaneous cancer model for human studies was already clearly recognized. In fact, it helped demonstrate that the overexpression of glutathione-S-transferase π was correlated with the limited accumulation of cisplatin in cells in vitro and reduced survival of canine patients, partly due to the action of an ATP-dependent pump that would actively clear drug molecules complexed to the enzyme from tumor cells [16]. Initial comparative studies have shown that the complementary DNA of the gene encoding P-gp, and consequently its fundamental function of active efflux of potentially toxic xenobiotics from cells, show high similarity among vertebrates. Nevertheless, there are species-specific differences in the promoter regions that regulate gene expression. Indeed, canine P-gp showed a comparable function to that of humans, but differences in the downstream gene promoter suggest that its expression may be regulated differently in canine tumor cells, which overall supports interest in further investigations of this translational issue [17]. One of the first studies using the canine osteosarcoma cell line OS2.4 demonstrated that doxorubicin treatment was able to select clones resistant even to vincristine through the overexpression of P-gp, similar to the results obtained in human osteosarcoma cell lines and biopsy specimens [18]. These findings first suggested the utility of the canine osteosarcoma model to study MDR mechanisms in human counterparts. A subsequent clinical study, which performed an expression profile analysis on genes regulating cell proliferation, metastasis, and DR in canine osteosarcoma patients, found that MGST1 and HMGB1 genes were overexpressed in dogs with MDR as in humans with other tumors. Overall, MGST1 has been shown to be involved in the detoxification of cells from various cytotoxic drugs, while HMGB1 mainly regulates DNA damage repair and the cell cycle, with antiapoptotic effects [19]. These in vivo results were consistent with in vitro studies conducted using canine lymphoma and leukemia cell lines. The canine B-cell lymphoma cell line GL-1, exposed to doxorubicin, developed a P-gp-mediated resistance even to vincristine, which was reversed by verapamil, while it remained sensitive to cisplatin [20]. Doxorubicin and vincristine are known to be involved in P-gp-related human MDR, which interferes with the accumulation of these drugs within cells. The P-gp function was inhibited by masitinib, a tyrosine kinase inhibitor, increasing the uptake of the substrate rhodamine123 (Rh123) and reversing doxorubicin resistance in the canine lymphoid cell line GL-40, a doxorubicin- and vincristine-resistant subclone selected from the GL-1 cell line after incubation with increasing concentrations of doxorubicin [21,22]. Indeed, the P-gp inhibitor PSC833 completely reversed resistance to chemotherapy and Rh123 uptake, while treatment with masitinib in the GL-1 cell line, which does not express P-gp, had no evident effects on doxorubicin-induced cytotoxicity [21,22]. Based on these results, the in vitro canine model, translationally comparable to human non-Hodgkin lymphoma, could represent a promising experimental system for screening P-gp-mediated DR.

Together with the molecular findings from the analysis of canine lymphoma samples [23], these results also suggest the opportunity to investigate the potential clinical utility of targeting P-gp to improve therapeutic efficacy in canine and human patients with MDR to cytostatic agents. However, for brain tumors such as glioblastoma, obstacles to achieving an effective drug concentration at the tumor site are represented not only by P-gp expressed on brain endothelial cells, but also by the limited permeability of the BBB. The drug efflux pumps MDR1 and BCRP1 are expressed on the luminal side of the brain capillary and, together with tight junctions between endothelial cells, form the BBB that limits the brain penetration of lipophilic drugs. The role of these efflux pumps on drug distribution in the CNS has been extensively evaluated using the experimental Madin–Darby canine renal epithelial cell line (MDCKII) system.

Interestingly, an initial experiment using MDCKII cells stably expressing human MRP1 and its variants in the N-terminal regions showed that this region, although highly evolutionarily conserved, would not be involved in the transport function of MRP1 [24]. Considering the crucial role of drug efflux transporters in the chemotherapy resistance of human tumors, this in vitro canine model highlighted the need for detailed analyses to define the role of different protein domains and provided preliminary useful data for the future design of clinically relevant inhibitor molecules.

By using MDCKII cells overexpressing P-gp, MRP1, or MRP2, HhAntag691 was shown to be both a Hedgehog (Hh) pathway inhibitor and an ABC transporter inhibitor, and to be able to reverse colchicine resistance. These findings suggest the potential use of agents simultaneously inhibiting Hh signaling and ABC transporters as promising anticancer drugs against cancer stem cells (CSCs) and brain tumors [25].

Similarly, the potential development of inhibitors of polymerases (PARPs), which play a key role in modulating DNA repair, has been studied in the same cell model overexpressing MDR1 or BCRP1, with the aim of synergizing conventional cytotoxic drugs [26].

Interestingly, the in vitro intracellular accumulation of rucaparib was significantly reduced in cells expressing both MDR1 and BCRP1 compared to control cells, while treatment with specific efflux pump inhibitors increased its uptake, demonstrating that this compound represents an efflux substrate for both pumps. Consistent with in vitro results, in vivo findings obtained using subcutaneous murine glioblastoma xenografts highlighted that rucaparib could potentially act synergistically with temozolomide, while the lack of the same effect in orthotopic glioblastoma mouse models confirmed that MDR1 and BCRP1 significantly limit the potential utility of PARP inhibitors in clinical use [26]. Related to this issue, a study on MDCK cells transfected with human P-gp, human BCRP, or mouse BCRP1 clearly indicated that the promising PI3K/mTOR inhibitor GDC-0084 was not a substrate of these ABC transporters [27]. These preliminary data suggested that the PI3K pathway, frequently altered in glioblastoma, could represent an interesting target for the treatment of brain tumors using agents that freely cross the BBB, laying the groundwork for the following preclinical studies and clinical trials. In a subsequent investigation, the MDCK-MDR1 cell line served as a useful in vitro model to test the ability of a novel iron oxide-based doxorubicin nanocarrier to cross the BBB and bypass the MDR. It effectively enhanced chemotherapy uptake in both this cell model and in U251 human glioblastoma cells, when combined with the transient opening of BBB tight junctions [28]. With the common goal of improving drug delivery into the brain, the same glioblastoma cell line and MDCK cells transfected with BCRP (MDCK-BCRP) were appropriately used to study the inhibitory effect of elacridar on P-gp and BCRP at the BBB level [29]. Elacridar accumulation in transfected cell lines was significantly lower than in wild-type cells, suggesting that P-gp and BCRP limit its uptake. Consistent with in vitro findings, the in vivo brain distribution of elacridar in mouse models was influenced by the active efflux exerted by P-gp and BCRP, which interestingly appeared saturable. This study highlighted that high concentrations of elacridar are necessary to saturate the transporters at the BBB, thus effectively improving the delivery of a chemotherapy drug into the brain and overcoming the DR in brain tumors [29].

Numerous other examples demonstrate the robustness of MDCK cells for translational studies of efflux transport in anticancer DR research.

MDCK-MDR1 and MDCK-BCRP cells supported preclinical and clinical evidence that osimertinib, a potent epidermal growth factor receptor (EGFR)-tyrosine kinase inhibitor (TKI), may be a promising therapeutic option in the management of brain metastases from non-small cell lung cancer. Although in vitro canine models confirmed that osimertinib is a substrate for the efflux transporters P-gp and BCRP, which commonly limit chemotherapy delivery to the CNS, leading to DR, it showed greater BBB penetration than conventional agents in murine models and patients [30,31]. These findings suggest interesting avenues for improving the efficacy of treatment against brain lesions in patients with acquired resistance to current EGFR-TKIs and highlight the utility of in vitro canine models for screening transporters in the development of new anticancer drugs. In the perspective of efforts to discover new P-gp inhibitors that counteract MDR, MDCK-MDR1 cells have been useful to test the P-gp inhibitor HZ08, highlighting its ability to reverse MDR when combined with vincristine or paclitaxel treatments both in vitro and in murine xenograft models [32]. Similarly, the canine MDCK cell line expressing BCRP2 was used to evaluate the activity of its quinazoline- and 4-methylpyrimidine-based inhibitors as potential tools to overcome MDR, by selecting the most promising compounds for in vivo applicability [33]. Recently, the MDR modulation in the clinical field was addressed by using canine MDCK cell lines for evaluating brivanib, a novel TKI, to circumvent transporter-mediated resistance combined with classical cytostatic agents [34]. In particular, cell sublines overexpressing human ABC drug efflux transporters significantly increased the uptake of non-cytotoxic probes as well as of mitoxantrone and daunorubicin upon brivanib exposure, partly reversing resistance to these cytostatic agents, without the apparent development of resistance to brivanib itself [34].

Another current challenge in the fight against DR is the development of novel compounds able to counteract tumor progression and recurrence by targeting P-gp expressed by cancer stem cells (CSCs) rather than acting primarily on cell proliferation mechanisms. In this regard, MDCK-MDR1, MDCK-MRP1, and MDCK-BCRP cell lines allowed the identification of a novel specific P-gp ligand among tetrahydroisoquinoline derivatives. This compound demonstrated a potency comparable to that of elacridar and further enhanced doxorubicin chemosensitivity. Importantly, the efficacy of this P-gp inhibitor was confirmed in human CSC models derived from patients affected by glioblastoma and malignant pleural mesothelioma [35]. In addition, the P-gp inhibitory activity of crown ether compounds, an emerging class of anticancer agents, was tested with the help of MDCK-MDR1 cells, contributing to the design of novel molecules to reverse P-gp-related MDR in tumors cells [36]. Continued efforts to overcome some safety and efficacy issues with P-gp inhibitors may help improve the targeting of P-gp-mediated MDR in the future. Of course, future in vivo investigations on MDR could take advantage of these in vitro findings. Similarly, using different canine osteosarcoma cell lines as an in vitro model, it has been demonstrated that MDR1 is a molecular mechanism related to resistance to the topoisomerase inhibitor etoposide, a member of a promising class of chemotherapeutic agents [37]. Human topoisomerase I, IIα, and IIβ have been recognized as potential targets for the development of novel anticancer inhibitory molecules for the treatment of diverse cancer types, and widely investigated in the updated literature. The relationship between the low expression of MDR1 and the clear susceptibility of cancer cells to topoisomerase inhibitors was supported by in vivo findings. In fact, the tumor growth in xenograft mouse models after the subcutaneous implant of these cell lines was markedly reduced by etoposide treatment, in contrast to tumors induced by injecting canine mammary gland cancer cell lines in which MDR1 expression was upregulated [37]. These results suggest the usefulness of further investigations on the role of MDR1 as a predictive biomarker to evaluate outcomes of treatment with topoisomerase inhibitors and MDR in comparative oncology. In further experiments, aimed at studying potential compounds capable of blocking the mechanism by which anticancer drugs are moved out of resistant tumor cells, MDCK cells and the variant expressing the human ABCB1 protein (MDCK-MDR1) were used to study the activity of isobavachalcone, a plant-derived molecule that has shown antiproliferative activity on several tumor cell lines [38]. Chalcone was shown to exert a higher cytotoxic effect on MDKC cells than on MDCK-MDR1 cells, suggesting its interference with the ABCB1 transporter. For validation, the accumulation of the ABCB1 substrate Rho 123 was found to be twofold higher in MDCK cells compared to MDCK-MDR1 cells. In addition, the chalcone treatment of MDCK-MDR1 cells combined with verapamil clearly reduced cell growth, confirming that it was a substrate of the ABCB1 protein. These results suggest that the survival rate of MDCK-MDR1 cells was reduced by transporter inhibition, which in turn increased the accumulation of this active molecule inside tumor cells [38].

### 3.2. Cancer Stem Cells (CSCs)

The proliferation of CSCs in the context of the tumor mass and their evident resistance to conventional chemotherapies contribute significantly to the development of DR and cancer recurrence in both canine and human patients. The CSC phenotype has been identified in cell populations of several canine and human tumors, including mammary, prostate, and hepatocellular carcinomas, leukemia, melanoma, glioblastoma, and osteosarcoma, offering valuable translational models of complementary relevance to induced or xenograft rodent models.

Therefore, the identification of CSC markers could aid in the development of novel translational therapies. Several comparative oncology studies have investigated the role of CSCs in canine and human osteosarcoma. Spontaneous canine osteosarcoma shows local invasiveness and frequent metastatic disease to lungs, with high tumor microenvironment heterogeneity and poor prognosis for treatment inefficacy, similarly to the human one. Conventional chemotherapeutics such as methotrexate, doxorubicin, and cisplatin are valid in both veterinary and human clinics for the standard treatment of osteosarcoma. However, at present, no new strategies are available for resistant or recurrent tumors in short-term follow-ups, making investigations into the cellular mechanisms involved in DR of fundamental importance. Osteosarcoma cells may develop DR by the impairment of drug uptake, enhanced DNA repair system activity, evasion of apoptosis, adaptive signaling from the tumor microenvironment, and the presence of CSCs. In particular, the involvement of a small fraction of CSCs in tumorigenesis, as well as in treatment failure, has been clearly observed in both naturally occurring canine and human osteosarcoma, and in this regard, similarities between canine and human cells have also been demonstrated [39]. Interestingly, Cyclooxygenase-2 (COX-2) overexpression was found in histological samples from both canine and human patients carrying osteosarcoma, suggesting a potential role of therapeutic strategies using COX-2 inhibitors. COX-2 is an inducible prostaglandin (PG) synthetase and PGs are likely to play a crucial role in angiogenetic and apoptotic processes in cancer. In this regard, COX-2 inhibition was shown to have no effect on the growth and DR of CSCs derived from both canine and human osteosarcoma cell lines, contrary to what was observed in daughter cells. Even though COX-2 expression was found to be upregulated in CSCs isolated from a canine patient with primary osteosarcoma, COX-2 inhibition using meloxicam and mavacoxib did not influence the growth and DR of CSCs in vitro, but it was able to prevent sphere formation from daughter cells, indicating a potential significant role for COX-2 in tumor initiation [39]. Similarly, the B-cell-specific Moloney murine leukemia virus integration site 1 (BMI1), a member of the Polycomb repressive complex 1 (PRC1) of transcriptional regulators, has been found to be highly expressed in tissue samples of primary and metastatic osteosarcoma from both dogs and humans. In particular, BMI1 seems to be involved in DR, suggesting that dog models may represent a valuable preclinical model to study the therapeutic potential of inhibition for this biomarker.

Indeed, BMI1 expression was found to be upregulated in both human and canine osteosarcoma cell lines, and its inhibition in vitro significantly reduced cell proliferation and increased sensitivity to carboplatin and doxorubicin in different canine lines. The overall results highlighted an interesting translational comparability between the tumor biology of human and canine osteosarcoma and a potential utility of targeting BMI1 in CSCs of this malignancy to overcome the challenge of DR in response to first-line chemotherapeutics [40]. The role of other transcriptional regulators such as hairy and enhancer of split-1 (HES1) regulators in modulating CSC maintenance and chemoresistance in osteosarcoma is still unclear. HES1 expression has been associated with increased aggressiveness in human osteosarcoma cell lines. The analysis of HES1 actions in primary canine osteosarcoma samples revealed that HES1 mRNA expression could be higher in tumor samples than in healthy bone tissue within individuals, while it was comparable to normal bone samples in dogs with faster tumor recurrence after treatment. HES1 expression was also found to be variable between cell lines in canine and human osteosarcoma cells. Overall, these findings suggest that other regulatory mechanisms may contribute to the aggressiveness of osteosarcoma and further research in this field may provide new insights into therapeutic resistance in both canine and human cancers [41]. The fundamental involvement of CSC-promoting mechanisms has also been demonstrated in canine and human gliomas. Canine gliomas resemble human gliomas in several clinical, imaging, and histological aspects, and similarities in various oncogenic signaling pathways have recently been discovered. The BMI1 protein has been found to be also involved in the development and DR of canine and human gliomas, through the generation of stem cells from astrocytes. The high expression of the BMI1 protein in glioma tissue samples from canine patients, as well as the antiproliferative effects of BMI1 inhibition by the PTC-209 molecule in several cell lines cultured from spontaneous canine gliomas, has been clearly evidenced, supporting the hypothesis of a potential utility of comparative studies to test new therapies, with benefits for both species. These findings suggest that spontaneous canine gliomas may represent an advantageous translational model, overcoming challenges related to experimental systems induced in laboratory rodents, for example to study novel treatments that can cross the BBB within a human-like vascular tumor microenvironment [42]. The molecular characterization of primary cultures of CSCs isolated from post-surgery canine osteosarcoma specimens as well as by immunohistochemistry from tumor fixed sections, has highlighted analogies with humans on the overexpression of chemokine receptor type 4 (CXCR4) and its chemokine ligand type 12 (CXCL12) [43]. This intracellular signaling pathway seems to be involved in osteosarcoma CSCs proliferation and migration in metastatic sites in both dogs and humans. By acting on this mechanism, the oral hypoglycemic drug metformin has been shown to increase the cytotoxicity of a combined treatment with doxorubicin and cisplatin in an in vitro canine osteosarcoma model, similar to the results described on human osteosarcoma cell cultures [43]. Considering that similar results have also been reported in stem cells derived from canine mammary carcinoma, human hepatocellular carcinoma, or human glioblastoma, the CXCR4/CXCL12 axis could represent a promising target to overcome the problem of DR, and results obtained in veterinary oncology may represent a preclinical starting point for the future improvement of human therapeutic strategies. The impact of metformin on the mechanisms of resistance to anticancer drugs has also been studied on a canine model of B-cell lymphoma, highlighting its role on the activity of the Anaphase Promoting Complex (APC), a molecular pathway evolutionarily conserved in living organisms. The APC has been found to be altered in dogs carrying DR lymphomas and in vitro using doxorubicin-resistant canine lymphoma cell lines. After the administration of metformin in combination with the conventional protocol including cyclophosphamide, doxorubicin hydrochloride (hydroxydaunorubicin), vincristine sulphate, and prednisone, canine patients with recurrent DR lymphomas showed decreased MDR proteins comparable to those that were found to be elevated in many cases of MDR cancers in humans, including MDR-1. Furthermore, APC activation was observed, leading to partial remission and clinical benefits, and this effect was confirmed by restored chemosensitivity using in vitro tests [44]. This canine model suggested that impaired APC activity may be a marker of MDR associated with poor prognosis, and that APC may represent a promising target in the prevention of anticancer DR in patients. Metformin has also been shown to suppress CSC survival in vitro as well as in preclinical models of breast cancer [45]. Spontaneous mammary tumors have high incidence in female dogs, representing a valuable opportunity for comparative oncology research, given the genetic, environmental, clinical, and molecular similarities to human breast cancer. In this regard, CSCs isolated from canine mammary carcinoma showed molecular characteristics and resistance to antineoplastic agents, similar to their human counterpart. Compared to continuous cell lines, these CSCs derived from spontaneous, non-treated canine mammary cancer advantageously represent the tumor biology and heterogeneity in vivo, even when implanted in mouse models, thus improving the knowledge provided by experimentally induced cancer in laboratory rodents. Overall, these aspects strongly spotlight the translational validity of new anticancer drugs tested in preclinical models and show that their optimization improves the identification of clinically useful compounds in humans. CSCs derived from canine mammary tumors have shown resistance to doxorubicin, mainly related to the involvement of ABC transporters in limiting the intracellular accumulation of the drug, as demonstrated by their inhibition by verapamil. Conversely, metformin significantly reduced CSC proliferation both in vitro and in immunodeficient xenograft mice models, as assessed by the Kiel 67 antigen Labelling Index (Ki-67-LI), a proliferation marker for several human and canine cancers [45]. These findings could be helpful in developing successful therapeutic strategies targeting breast cancer CSCs for the benefit of both veterinary and human medicine. Similarly, canine prostate cancer closely resembles human prostate cancer in clinical progression and histopathological features, and CSCs play a pivotal role in its therapeutic resistance. Preliminary in vitro studies using the canine prostate adenocarcinoma cell line CT1258 show mild doxorubicin resistance, high metabolic activity, and the expression of the alpha-6 integrin that characterizes tumor cells with stem-like characteristics. These findings represent promising translational information to further characterize the role of CSCs in prostate cancer and discover potential therapeutic targets through in vivo studies [46]. Recently, the sex determining region Y-box 2 (SOX2) has been identified as a crucial regulator of CSCs pluripotency in different tumors of humans and rodent models and has been therefore correlated to DR and poor prognosis. In a comparative oncology investigation, SOX2 overexpression was also found in tissue samples from many types of canine neoplasia. Although the role of this CSC marker in each tumor histotype remains to be better understood, these findings may provide useful insight into DR mechanisms and help guide new translational perspectives to address this relevant challenge in comparative oncology [47].

Among the new generation of targeted therapeutic strategies to overcome the intrinsic or acquired resistance of tumor cells to conventional cytotoxic agents or radiotherapy, bispecific ligand-targeted toxins (BLTs), designed to bind specific receptors expressed by tumor cells, have been employed, with limited toxic side effects.

Interestingly, highly chemotherapy-resistant Emma, Frog, and SB cells derived from canine hemangiosarcoma, including CSC-enriched cultures, have demonstrated an improvement in cytotoxicity and the safety of a newly synthesized deimmunized Pseudomonas exotoxin conjugated to epidermal growth factor and urokinase (EGFuPA toxin), which are overexpressed in several tumors and particularly in sarcomas and tumor endothelial cells.

Canine hemangiosarcoma represents a suitable model to study highly resistant sarcoma, which is very similar from a molecular point of view to human angiosarcoma and, in particular, a tumor stem cell platform in which CSCs play an important role in contributing to chemoresistance [48]. Overall, these data highlighted the utility of the canine hemangiosarcoma models to develop novel approaches to circumvent DR, suggesting integrative fields for cancer treatment, particularly in tumors where increased resistance to conventional cytotoxic drugs has been associated with CSCs.

### 3.3. Epigenetic Alterations

Epigenetic modifications of DNA, such as methylation, histone modification, or chromatin remodeling, can upregulate the expression of oncogenes, induce increased drug efflux, enhance DNA repair, or impair apoptosis, resulting in DR. Methylation has been linked to chemoresistance in canine lymphoma, making this animal model an attractive candidate for testing demethylated drugs for use in both chemotherapy-resistant canine and human patients. Among drug-resistant cellular methylation models, the hypermethylation of the CpG island in the region upstream of exon 2 in the ABCB1 gene was found in drug-sensitive canine lymphoma cell lines, in contrast to hypomethylation in drug-resistant ones, as confirmed in vivo. Similarly, the methylation of other gene promoter regions has been found to be associated with resistance to L-asparaginase (L-asp) [49], as well as to lomustine or doxorubicin [50], in canine lymphoma cell lines. In particular, the canine lymphoma cell lines OSW and CLGL-90 have been useful in further elucidating the mechanisms related to L-asp resistance in lymphoma, demonstrating that the epigenetic regulation of asparagine synthetase expression by the methylation of its promoter was inversely related to mRNA or protein levels and directly to L-asp resistance [49]. However, asparagine synthetase methylation showed a variable status in canine patients with high-grade B- and T-cell lymphoma and was not significantly correlated with clinical outcomes. Furthermore, the asparagine synthetase promoter was found to be overall hypomethylated in both human lymphoma cell lines and tissue samples. Such contradictory results between in vitro and in vivo data suggest the need for further studies to evaluate the utility of L-asp in the translational therapy of lymphoma [49].

### 3.4. Apoptosis and Epithelial–Mesenchymal Transition (EMT)

Cancer cells are more resistant to apoptosis, a form of cell death triggered by exposure to various cellular stresses. This biological characteristic not only allows the tumor to become more aggressive but also contributes to DR. Exploring the scientific literature, the canine model seems particularly relevant to study aberrations in gene expression involved in antiapoptotic mechanisms and associated with DR in both humans and dogs. Furthermore, some reports of potential therapies targeting these mechanisms in both species suggest their possible efficacy and prospects for future research. B-cell lymphoma is the most common hematopoietic malignancy in both dogs and humans and shares many biological, genetic, and molecular characteristics, including the Feline McDonough Sarcoma (FMS)-like tyrosine kinase 3 (FLT3) mutation. In a constitutively active state, this oncogene targets several downstream proteins such as the signal transducers and activators of transcription (STAT), mitogen-activated protein kinase (MAPK), and protein kinase B (AKT) pathways, leading to cellular proliferation and resistance to apoptosis [51]. Using genomic polymerase chain reaction (PCR), conserved FLT3 mutations between species have been demonstrated in both the canine GL-1 cell line and in clinical specimens of canine B-cell lymphoma. Although FLT3 mutations found in canine models show differences in frequency of occurrence and prevalence in distinct histotypes compared to the human counterpart, the major downstream-targeted pathways, STAT5 and extracellular signal-regulated kinase (ERK)1/2 proteins, appeared overall evolutionarily conserved. Furthermore, the canine GL-1 cell line was found to be sensitive to the FLT3 inhibitor lestaurtinib, similar to the human leukemia cell line MV4-11 carrying a comparable FLT3 mutation [51]. Overall, these findings highlight that both cellular and clinical canine models of leukemia may be appropriate to study common mechanisms of oncogenesis relevant to both species. FLT3 appears to be a promising therapeutic target, in light of its upregulation in several forms of acute leukemia, and a deeper understanding of FLT3 mutations through valid experimental models could improve the development of more effective FLT3 inhibitors in overcoming DR. Similarly, the activation of the B-cell nuclear factor kappa-light-chain-enhancer (NF-kB) pathway has been shown to play a central role in MDR by promoting cell proliferation and exerting antiapoptotic effects. In companion dogs with spontaneous B-cell lymphoma, the comparative overexpression and constitutive activity of NF-kB have been found [52]. Furthermore, the intranodal administration of the IK kinase (IKK) complex-selective peptide inhibitor of the essential modulator-binding domain of NF-kB (NEMO) in canine patients with relapsed B-cell lymphoma was able to inhibit NF-kB expression and reduce tumor burden. In vivo findings, together with the in vitro induction of apoptosis in primary malignant B cells, highlighted for the first time the translational relevance of NF-kB inhibition in the treatment of B-cell lymphoma [52]. In a following study, improved methodologies were implemented to work with canine primary cells, including optimized procedures for isolating lymphocytes from canine malignant lymphoid tissue, treating them with novel anticancer drugs, and assessing signaling pathways relevant to proliferation, survival, and DR. Taking advantage of these advances, the therapeutic potential of the NEMO-binding domain peptide was studied in primary canine malignant B cells in vitro, showing the ability to induce apoptosis by blocking NF-κB signaling via the inhibition of the upstream IKK regulatory complex [53]. Overall, available data of aberrant NF-kB signaling in different canine cancers, and on the potential to inhibit NF-kB activity by using antineoplastic compounds, suggest that dog models may be a suitable model for comparative cancer biology studies on the regulatory mechanisms of NF-kB leading to DR in human cancers [54]. Spontaneous canine mammary tumors show a clinical evolution and molecular features similar to those of human breast cancer. Canine mammary gland cancer cell lines appear to be of great value for translational research on the tumor microenvironment, treatment, and DR in breast cancer. The canine mammary cancer cell line B-CMT established from a primary mammary adenocarcinoma diagnosed in a female dog, has been described as a triple-negative cell line that overexpresses hypoxia inducible factor-1α (HIF-1α), leading to doxorubicin resistance by the inhibition of apoptosis and P-gp overexpression. Furthermore, the B-CMT cell line appears to be able to replicate the biological characteristics of the primary tumor in immunodeficient mouse xenografts [55]. These features could be of great significance to provide further insights into future research, including that on MDR. Recently, the effect of celastrol, an extract of the plant Tripterygium wilfordii, an alternative treatment in traditional Chinese medicine for cancer, was comparatively studied on canine mammary tumors, aiming to improve treatment options in canine and human patients [56].

In particular, celastrol has been shown to inhibit NF-kB and B-cell leukemia/lymphoma 2 (Bcl-2) signaling pathways, as well as increase the expression of the Bcl-2-associated X (Bax) protein and caspase, involved in the induction of the apoptosis of several types of tumor cells, including breast cancer. The in vitro proliferation and migration ability of the triple-negative canine mammary gland cancer cell line CMT-7364, as well as cell line CIPp, were selectively inhibited by celastrol treatment in a dose-dependent manner, acting through a dual mechanism by inducing caspase-mediated cell apoptosis and cell cycle arrest by regulating cell cycle-related proteins. Considering that consistent results were found in human triple-negative breast cancer cell lines, this study provided a proof of concept on the anticancer mechanisms of celastrol and for its potential clinical application [56].

Gene expression analysis in primary human tumors has emerged to identify biomarkers of chemotherapy resistance and pathways related to treatment response. In this regard, the comparative analysis of gene expression profiles has highlighted marked similarities between human and canine osteosarcoma. In particular, a molecular screening of poor- versus good-responder canine osteosarcoma patients revealed that the Hh signaling pathway plays a crucial role in both osteosarcoma progression and therapy resistance, primarily through the inhibition of apoptosis [57]. Although the overall mechanisms by which Hh signaling promotes DR are still under investigation, understanding them is relevant to the development of effective treatment strategies. The results of this study suggested the potential usefulness of comparative genetic and molecular prognostic screening between humans and dogs to translate novel insights into the mechanisms of osteosarcoma progression and chemoresistance. More recently, enhanced cell proliferation and reduced apoptosis, leading to DR, were comparatively found in several human and canine osteosarcoma cell lines related to the overexpression of ephrin A2 (EphA2) receptor tyrosine kinase. The upregulation of EphA2 in both human and canine osteosarcoma cells supported migration and invasiveness in vitro, likely mediated by integrin β3 expression, as confirmed by EphA2 silencing [58]. Consistent with these findings and based on the same comparative oncology approach, the suppression of EphA2 activity was found to significantly reduce tumor growth in xenograft mouse models of implanted canine osteosarcoma cells. Furthermore, increased EphA2 expression induced the resistance of osteosarcoma cells to cisplatin in both species, probably through the activation of the Rous sarcoma proto-oncogene (cSRC) and AKT and/or ERK–MAPK pathways, as confirmed by the reduction in their phosphorylation following EphA2 silencing [58]. Overall, the similarities in EphA2 receptor expression and function in both species suggest that it may represent a promising therapeutic target both to overcome MDR in osteosarcoma and to potentially reduce the side effects of cisplatin in combination therapies. The translational potential of these findings could benefit from the shorter lifespan and faster progression of osteosarcoma in canine patients compared to humans, improving knowledge of treatments with benefits for both species. Although there are numerous similarities in the mechanisms of DR in various canine and human tumors, it is important to recognize that species-specific differences have also been found. Aurora kinases A and B (AURKA, AURKB) are involved in cell mitosis and are overexpressed in several human malignancies, thus representing promising therapeutic targets. Some canine osteosarcoma cell lines highly express Aurora kinases, but treatment with their inhibitors failed to consistently increase apoptosis compared to the effects exerted on human tumors that overexpress these kinases, highlighting differences in the potential utility of these drugs in veterinary and human cancer therapy [59]. In tumor cells, telomerase activity and the maintenance of telomere stability are associated with increased cellular resistance to apoptosis. For this reason, several antitumor strategies have been studied that inhibit the telomerase enzyme, causing DNA damage and programmed cell death in experimental models. In this regard, the effect of telomerase inhibition by small interfering RNA (siRNA) and short hairpin RNA (shRNA) oligonucleotides was evaluated in canine hemangiosarcoma SB/HSA.2 cells [60]. The study results suggested that telomerase could be a potential target for anticancer therapies in dogs, but measurements of telomerase expression also showed that these cells develop resistance to the inhibition of this enzyme over time. Subsequent in vivo tests in murine xenograft models of canine hemangiosarcoma inoculated with early- or late-passage SB/HSA.2 cells demonstrated that telomerase inhibition could effectively target the growth of canine hemangiosarcoma, but there is also the possibility that resistance may occur. Overall, these findings suggest that natural canine cancer models may be useful in bridging the translational gap between human patients and mouse models in the study of telomerase-based therapies [60].

Epithelial–mesenchymal transition (EMT), a cellular process that tumor cells can undergo, plays a crucial role in tumor recurrence and DR-related metastasis, representing a relevant clinical problem in prostate cancer therapy. Several regulatory mechanisms are involved in EMT, and resistance to the apoptosis of cells detached from the surrounding extracellular matrix is among the mechanisms through which EMT may contribute to anticancer DR. Spontaneous canine prostate cancer has proven to be a valuable model of androgen-independent prostate cancer in humans, as well as the canine Ace1 cell line, derived from a primary canine prostatic carcinoma, which shares similar signaling pathways and receptors upregulated in the human counterpart. Using this cell model, AR-42, a promising histone deacetylase inhibitor, was shown to reduce tumor cell proliferation in vitro primarily by inducing apoptosis, and the in vivo intracardiac injection of Ace-1 cells into nude mice demonstrated that AR-42 was able to prevent the bone metastasis of prostate cancer [61]. In this perspective, future studies evaluating the effects of new histone deacetylase inhibitory anticancer drugs on other human and canine prostate cancer cell lines could be useful to develop therapies more effective to target EMT- and apoptosis-related resistance, in turn counteracting prostate cancer bone metastasis.

The overexpression of epidermal growth factor 2 (HER2) in women, together with the lack of steroid hormone receptors, is associated with more aggressive breast tumor growth and the acquisition of DR through major pathways such as the wingless-type MMTV integration site (Wnt) family or the Rous sarcoma proto-oncogene (cSRC). Interestingly, the canine mammary tumor (CMT)-U27 cell line showed high basal Wnt activity and high levels of EGFR, HER2, and HER3 mRNA expression, similar to HER2-overexpressing human luminal cell lines. These cells are characterized by an EMT phenotype, suggesting that the inhibition of Wnt activity could influence breast cancer malignancy in some histotypes. Indeed, the inhibition of Wnt activity led to the reduced invasiveness of CMT-U27 cells. Furthermore, highly activated Wnt signaling in the canine mammary cell line CMT-U27 was not found to correlate with HER2/3 signaling by siRNA silencing, nor with cSRC activation, as demonstrated by in vivo experiments using the CMT-U27 xenograft in zebrafish embryos treated with the cSRC inhibitor dasatinib [62]. These findings suggest that CMT-U27 is a valuable comparative model, particularly for mechanistic studies and targeted drug screening involving the HER2 and Wnt pathways, but should be used in combination with human breast cancer models to validate the findings prior to clinical translation, due to significant interspecies and specific biological limits. Further studies are needed to elucidate the complex interaction of signaling pathways in DR to HER inhibitors in HER2/3-positive breast cancer.

### 3.5. Drug Target Alteration

Compared to conventional cytotoxic agents, selective targeted therapies have the advantage of interfering with tumor growth by inhibiting the activity of specific proteins with fewer side effects on normal cells.

Several genetic mutations involving chemotherapeutic targets, such as tyrosine kinases and topoisomerases, lead to therapeutic resistance through alterations in molecular signaling pathways or binding.

Recent advances in genomic and proteomic analyses have contributed to their increasing identification in both humans and canine species.

Over the past decades, these mechanisms have been studied fragmentarily in various canine cell lines and in natural tumors.

Signal transduction between the HER1 and HER2 transmembrane tyrosine kinases and cell nuclei is mediated by RAS proteins, and mutations in its gene KRAS are associated with resistance to conventional monoclonal antibodies that target those proteins. The role of this molecular pathway has been widely recognized in human gastric cancer and, interestingly, HER1 and HER2 were found to be overexpressed in several canine gastric tumor samples. Although the DNA sequence of the canine KRAS gene is very similar to that of humans, KRAS mutations were uncommon in the canine patients analyzed. Nevertheless, a single mutation of codon 12 is the most frequent in humans and could similarly determine DR in dogs by altering the inhibitor/target interaction and preventing binding [63]. Although gastric tumors in dogs closely resemble human clinical and histological features, their incidence is rare.

Further studies on the role of HER1/HER2/KRAS pathways in canine oncology could help to understand the utility of this comparative model to address DR in both canine and human patients [63]. Instead, a comparative analysis of melanoma cell lines derived from primary tumors or the metastasis of human, canine, and equine species showed comparable mutations in proto-oncogenes BRAF, NRAS, and KIT, which are well-known factors regulating proliferation and apoptosis in human melanoma [64]. Indeed, the inhibitor LY3009120, which targets these pathways in both cells with and without RAS mutations, has been proposed as a promising therapeutic approach in comparative oncology, with the advantage of the minimal paradoxical activation of ERK signaling compared to BRAFV600E-selective inhibitors, thereby reducing the risk of unwanted enhanced tumor growth. Significant growth inhibition was observed in nearly all melanoma cell lines after exposure to LY3009120, suggesting its value for further in vitro and in vivo testing to target BRAF-resistant tumors and to study resistance mechanisms in comparative oncology [64]. Interestingly, the sequencing analysis of BRAF in canine transitional carcinoma cells revealed a close homology to human BRAF and that its mutations commonly result in increased MAPK pathway activity in both human and canine tumors. In addition, the upregulation of receptor tyrosine kinases has been found among the mechanisms of resistance to BRAF inhibition. In this regard, the in vitro growth of several BRAF-mutated canine transitional carcinoma cell lines was not inhibited by the BRAF inhibitor vemurafenib, whereas MAPK inhibitors do so similarly to human BRAF-mutant cell lines. However, the monotherapy of such cell lines with BRAF or MAPK inhibitors leads to a temporary attenuation of MAPK pathway activity, while the combination treatment with EGFR receptor inhibitors has been shown to act synergistically [65]. Taken together, these data suggest the utility of canine transitional carcinoma as a model to investigate the role of the MAPK pathway in modulating cancer progression and acquired DR.

Similarly, canine B-cell lymphoma has been proposed as comparative model of homologous human disease, with the advantage of an immunosuppressive tumor microenvironment over immunocompromised murine xenograft models. DR occurs in both species; therefore, dogs with spontaneous B-cell lymphoma could offer insights into immunotherapy and for identifying mechanisms of resistance to chimeric antigen receptor (CAR)-T-cell therapies [66]. Resistance to CAR-T-cell therapies occurs in most human patients by the loss or downregulation of CD19 and/or CD22 on malignant B cells, leading to “antigen escape”, or the expression of inhibitory ligands, such as programmed cell death 1 ligand 1 (PDL1), impaired apoptosis, and an immunosuppressive tumor microenvironment [66]. Overall, it would be beneficial to study these mechanisms using appropriate experimental models to develop more effective therapeutic strategies. The development of tumor resistance to CAR-T cells that target a single antigen is very common. A methodological study on the implementation of CAR-T-cell therapy in canine patients demonstrated that canine T cells, modified to express a chimeric antigen receptor specific for CD20, were able to cause cell death in canine and murine B-cell lymphoma cells expressing canine CD20 [66]. By critically considering the notable differences in reactivity between species to antigens, canine models could contribute to advance the knowledge on the mechanisms of adoptive immunotherapy and, in general, on cancer resistance [67].

Still, canine mast cell tumors have been suggested as a useful model of the altered function of Kit, a receptor tyrosine kinase present in various human and spontaneous canine cancers, for evaluating the efficacy of new inhibitory molecules [68]. Mutations in Kit have been found to induce its constitutive activation, promoting uncontrolled cell proliferation and survival. Furthermore, secondary mutations after monotherapy with specific inhibitors frequently lead to DR in the clinical setting, for example toward imatinib, sunitinib, or dasatinib, due to altered drug binding and/or activity. The expression of this oncogenic protein is regulated by heat shock protein 90 (HSP90); therefore, its targeting with inhibitors would degrade this protein and act as a valuable antitumor strategy.

In this regard, the effect of STA-9090, a heat shock protein (HSP) 90 inhibitor, was evaluated in canine malignant mast cells with different mutations of Kit. STA-9090 reduced the proliferation and viability of malignant mast cell lines as well as malignant mast cells cultured from histological samples, and it was able to inhibit tumor growth in vivo in a murine xenograft model [68]. This study suggests the opportunity for future clinical studies to test novel treatment strategies using HSPs inhibitors in canine patients with spontaneous cancers, to gain new insights useful to clinical application in humans.

### 3.6. DNA Damage and Repair

Among the different mechanisms of resistance to therapy developed by tumor cells, one of the most important is intratumoral heterogeneity. DNA repair pathways play an important role in maintaining genome stability, and defects in related mechanisms contribute to tumor heterogeneity, leading to the selection of cellular sub-clones, mutations, and resistance to single-agent chemotherapy. Chemotherapeutics that target DNA repair pathways have been explored; however, resistance to these drugs is also being found and studied and represents a growing problem in the clinical setting. In particular, osteosarcoma shows comparable genetic complexity and histotypic heterogeneity in humans and dogs, which favor recurrence and DR; therefore, affected individuals still show limited survival despite scientific advances in cancer treatment.

Therefore, osteosarcoma represents a promising candidate for personalized medicine approaches, and dogs have proven to be a valuable translational model for this cancer, helping to fill gaps in the knowledge of DR and identify potential therapeutic targets for future clinical trials. Canine patients with spontaneous osteosarcoma usually undergo limb amputation prior to chemotherapy, allowing for comparisons of the genetic profile between normal bone and tumor tissues sampled in the same subject and avoiding interpretation problems that occur in human patients related to acquired DR. In this regard, several human homologous genes are consequently upregulated by comparing their expression in primary canine osteosarcoma and normal tissues collected from the same chemotherapy-naïve subject. Among them, the GTSE1 gene was identified, which regulates the cell cycle and may induce cisplatin resistance in human osteosarcoma through the inhibition of p53, in turn blocking apoptosis in tumor cells in response to DNA damage [69]. More recently, the same research group conducted another comparative study on osteosarcoma, focusing on the role of tumor heterogeneity in the evolution of different cellular subsets that promote DR.

A primary canine osteosarcoma specimen before any therapy was characterized, showing significant genomic instability and chromosomal copy number variations, as well as clusters of osteoblasts, fibroblasts, endothelial cells, myeloid cells, and CD4+ T cells. These findings were consistent with those found in human patients, suggesting useful interspecies similarities in osteosarcoma heterogeneity [70].

## 4. Discussion

The complexity of cancer makes it advantageous to use a multidisciplinary approach and the combination of different state-of-the-art study tools and models in patient care.

Despite ongoing efforts to develop effective chemotherapeutics, DR in cancer currently represents the major limitation to patient survival.

Hence, the development of new treatment strategies overcoming MDR is an urgent need.

Canine cancer models have the advantage of representing a genetically broader population than inbred mouse models, and of sharing similar responses to the same chemotherapeutics as humans, better representing the heterogeneity and complexity of spontaneous human cancers, and addressing some scientific questions related to preclinical drug development and cancer resistance [10,67].

The literature on the mechanisms of DR in dogs with cancer and those shared with humans consists primarily of case reports scattered throughout the literature.

Therefore, we provided a comprehensive review of all currently known DR pathways found in common between humans and dogs with cancer, possible strategies to overcome them by targeted therapies, and a reflection on future perspectives.

The purpose of this review was to evaluate the potential utility of DR overlap between dogs and humans, providing evidence of the value of the canine model in translational research. The results of the reviewed studies showed that both canine cancer cell lines and dogs bearing spontaneous tumors represent powerful and still underutilized experimental models to develop new knowledge on chemotherapy and related DR [10].

From the past until today, most cancer research has been conducted on mouse models, mainly because of their easy handling and genetic manipulation, and relatively low breeding costs.

However, they have shown some limitations in mimicking human tumors, resulting in only a small fraction of the new drugs tested progressing to clinical trials and being approved for use in patients [67].

Overall, the use of murine and canine models of induced or spontaneous tumors, respectively, could be balanced to answer specific scientific questions in cancer research, but both have inherent limitations and ultimately need validation by human clinical trials. Connecting animal and human medical research outside the laboratory, in a One Health perspective, can help strengthen collaboration and coordination in cancer investigation.

To promote translational studies, it is important to highlight the relevance of experimental results and share them among researchers from different fields of life sciences.

Furthermore, citizen science projects, which involve the public in canine scientific research, could usefully integrate data collection into established experimental methods.

The bibliometric analysis of scientific articles selected from our literature search revealed a clear increase in research about the DR mechanisms shared by canine and human cancer patients over the years.

**Figure 3:**
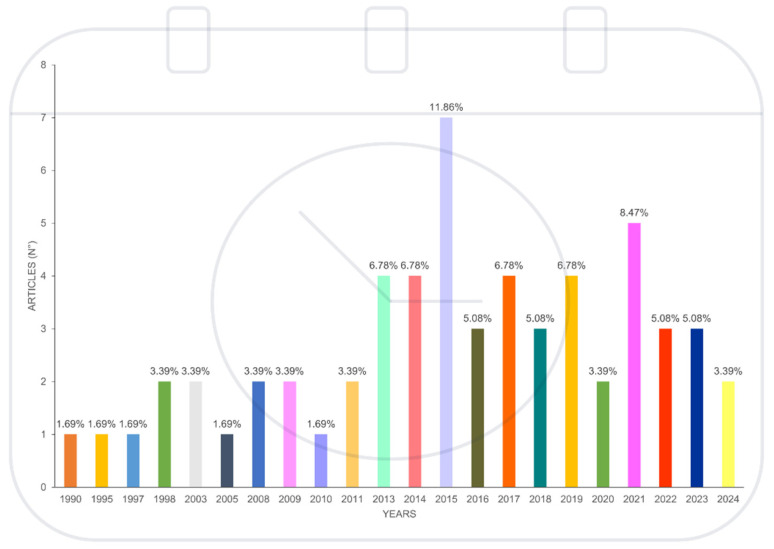
Annual scientific production.

Overall, Figure 3 illustrates the annual scientific production on the DR mechanisms shared by canine and human cancer patients from 1990 to 2024.

**Figure 4:**
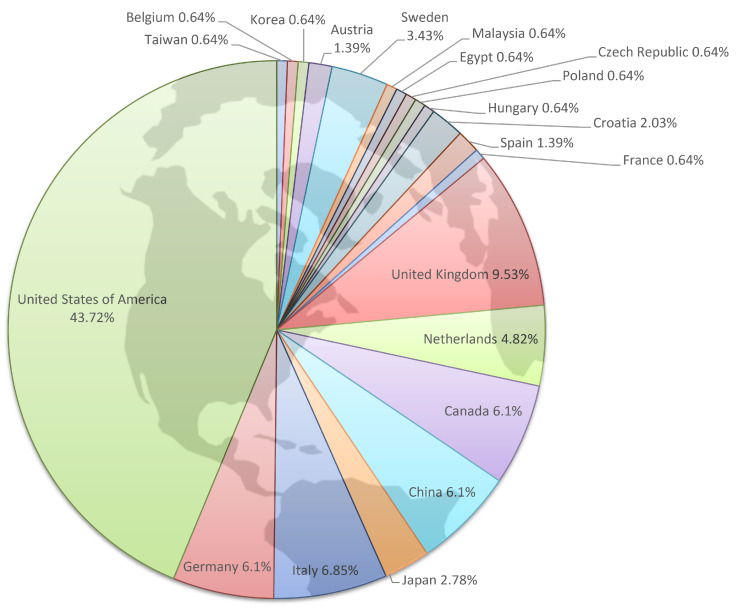
Scientific production by country.

Initially, from 1990 to 2000, the number of articles published annually on this topic remained relatively low, fluctuating between one and two articles per year. A notable increase begins in 2013 and continues until 2024, with the number of articles on this topic consistently reaching four articles per year and peaking in 2015 and 2021, with seven and five articles per year, respectively. Overall, these data demonstrate a growing interest in this topic, which translates in an increased research output, although in recent years, the COVID-19 pandemic has likely shifted the focus of One Health research away from comparative oncology. Several countries have shown an increasing sensitivity to the One Health approach in oncology, as highlighted in Figure 4, where scientific production by country is expressed as a percentage of the total articles found.

The United States is actively working on One Health approaches in oncology, with 43.72% of articles, followed by the United Kingdom with 9.53% of articles, in line with the historical leadership that these countries have had in the progress and ethics of veterinary medicine. Italy has also demonstrated a strong involvement in this research area, covering 6.85% of the articles. Germany, Canada, and China contributed almost equally with 6.1% of the articles, demonstrating significant participation in research on this topic. Netherlands, Sweden, and Japan have produced articles in a range from 4.82 to 2.78% and overall, another 12 countries across Europe, Africa, and Asia have produced articles in a range from 2.03 to 0.64%, underlining the widespread international commitment to advancing knowledge and addressing oncology issues through a “One Health” approach.

The study of spontaneous tumors in companion dogs has been advocated as a valuable opportunity for research into new therapies, with benefits for veterinary and human oncology. Advances in the genomic characterization of numerous types of canine tumors have facilitated the implementation of precision medicine to treat dogs. Importantly, given the natural occurrence of tumors in dogs sharing the human environment, the intra- and inter-individual complexity of cancer and resistance patterns can be more adequately represented compared to traditional research models [67].

Furthermore, some homologous tumors, which can be extremely aggressive and highly resistant to conventional therapies, occur spontaneously in companion dogs at a higher frequency than in humans.

This could have important clinical implications, providing an alternative source of valuable samples for comparative studies on challenges faced by humans.

Although dogs represent an attractive model for DR research, complementary to laboratory rodents, some limitations have been recognized. Species-specific differences in drug metabolism, molecular targeting, and chemoresistance mechanisms are considered critical for clinical extrapolation to humans. For example, canine and human species show similarities in hepatic cytochrome (CY) P450 isoforms, but differences in their metabolic pathways and substrate specificity, as well as inter-individual genetic polymorphisms, have also been described, which can influence the metabolism, excretion, toxicity, and dosing profiles of drugs, including chemotherapeutics [71]. The relevance of CYP450 enzymes as potential therapeutic agents against cancer, as well as a mechanism of drug resistance through the deactivation of anticancer drugs, has recently been highlighted [72].

For these reasons, the direct translation of findings predicting drug efficacy, potential side effects, and chemoresistance from the canine models to humans and vice versa requires careful consideration of species-specific factors.

An additional challenge for research and clinical translation purposes is the need to improve the standardization of tumor material collection, as well as to implement molecular analyses on large pools of canine breeds, to broaden the knowledge of the genetic landscape, resulting in further strengths for precision medicine. Interestingly, there are relative ethical concerns in treating canines with naturally occurring cancers compared to induced cancer models, and simpler regulations in veterinary medicine, including an informed owner’s consent, allows for the easier testing of modified conventional chemotherapy protocols and off-label drugs [73].

In particular, the One Health concept provides an integrative perspective on public health, but still carries with it an intrinsic criticality from an ethical and regulatory point of view and is currently finding its place in legislation.

The “One Medicine, One Health” approach is based on the idea that human and veterinary medicine should contribute to each other.

Inevitably, the status of “sentient beings” recognized in animals by European legislation influences the One Health vision.

However, a utilitarian and anthropocentric perspective continues to be mainly supported, rather than promoting the One welfare vision on the direct and indirect benefits of improving animal wellbeing, focusing on the prevention of pandemics, antimicrobial resistance, zoonoses, and emerging infectious diseases [74,75]. Nowadays, the concept of One Health has become much broader and could benefit from the integration of comparative oncology, translating knowledge on cancer resistance mechanisms into new prevention and treatment strategies in human patients [76]. In this perspective, the inclusion of translational studies on spontaneous canine tumor models in the preclinical evaluation of new anticancer therapies has been proposed as “proof of principle” investigations. However, several challenges hinder the effective implementation of the “One Medicine, One Health” strategies, including the still limited communication between veterinary and human health professionals, as well as other scientific researchers, and poor collaboration between interdisciplinary infrastructures [77]. Although veterinary clinical trials may be useful to improve drug development and address the problem of DR, taking advantage of the short lifespan of dogs and consequently the earlier evaluation of drug activity, their clinical validity has sometimes been questioned, due to the limited standardization of study designs.

**Figure 5:**
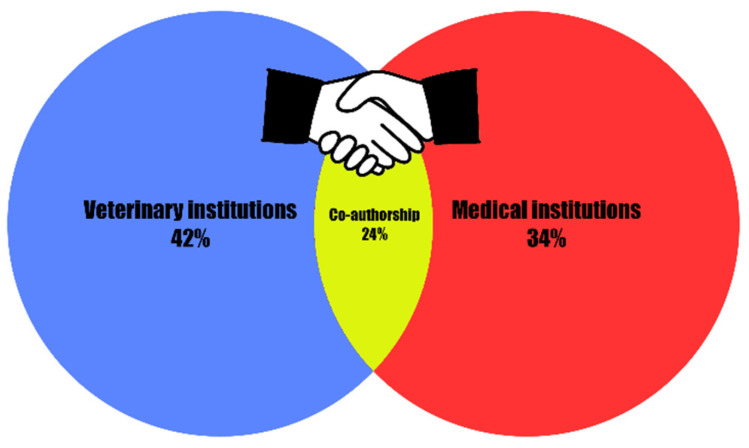
Authorship of scientific production.

This review shows that humans and dogs can complement each other in the study of cancer pathogenesis and therapy. A One Health approach, including human and canine patients, would represent a valuable joint effort and offer opportunities to accelerate knowledge, promoting the better survival of humans and dogs. The institutional authorship of the examined scientific production is shown in Figure 5 and expressed as a percentage based on the total number of affiliations found in the articles.

Veterinary clinical and research institutions provided the largest contribution (40.8%) compared to medical institutions; the latter mainly focused on evaluating the molecular mechanisms of DR in canine cellular models or using them to test new pharmacological compounds (34%). Interestingly, 24% of scientific research showed a co-authorship between medical and veterinary institutions, underscoring the importance of transdisciplinary cooperation in addressing complex health challenges such as chemotherapy resistance. Today, advances in comparative oncology knowledge and available diagnostic tools for early cancer detection in companion dogs, such as liquid biopsy, innovative imaging, and genomic and molecular testing, could enable broader translational benefits toward precision medicine across species. On the other hand, the economic accessibility and specialized expertise to these diagnostic technologies hinder their widespread application in veterinary oncology, comparable to the standards of human healthcare, with differences between countries around the world [78].

Furthermore, the cost–benefit ratio between welfare and life expectancy in dogs with cancer and concerns about the extra time and financial efforts required of dog owners, together with the acceptance of an uncertain success rate of therapies, may limit informed decisions about treatment modalities to palliative ones [79,80].

Therefore, implementing collaborative research activities with academic or community nonprofit organizations for cancer prevention and early diagnosis, coupled with policy efforts and comparative oncology training programs, could provide an interdisciplinary strategy to disseminate a cross-species utilitarian view and promote information on DR from natural tumor models for the benefit of humans and companion dogs.

## 5. Conclusions

Overall, we interpret these data to suggest that a deeper understanding of cancer biology in dogs and humans may be helpful in gaining the broader acceptance of preclinical natural models of canine cancer in the scientific community. They could valuably complement current induced rodent or xenograft models, addressing specific questions and ultimately benefiting both species. A conceptual framework for implementing a One Health approach in the study of comparative oncology and chemotherapy resistance should include legislation, funding, promoting future collaborations between human, veterinarian, and environmental institutions, linking the One Health vision to the 3Rs principle in animal experimentation, and pursuing a culture of care approach.

The implementation of the One Health approach highlights how progress for one species can also influence others.

We are confident that interactions between researchers studying human and animal health will become increasingly common and that collaborative efforts across disciplines will be encouraged, promoting scientific progress in accordance with the modern “One Medicine, One Health” perspective. We believe that initiatives that enhance interdisciplinarity will be useful in developing new treatment strategies for DR in cancer patients.

## Data Availability

The raw data supporting analyzed in this review are included in the article as Supplementary Materials.

## Supplementary Materials

The following supporting information can be downloaded at: https://www.mdpi.com/article/10.3390/cancers17122025/s1, Reference Management File (RM file).

## Abbreviations

The following abbreviations are used in this manuscript:

WOS	Clarivate Web of Science
MEDLINE	Medical Literature Analysis and Retrieval System Online
DR	Drug resistance
CCR	Cancer Institute’s Center for Cancer Research
COP	Comparative Oncology Program
OSF	Open Science Framework
ABC	ATP-binding cassette
P-gp	P-glycoprotein
MDR1	multidrug resistance 1
MRP1	multidrug resistance-associated protein 1
MRP2	multidrug resistance-associated protein 2
BCRP	Breast Cancer Resistance Protein
MDR	multidrug resistance
BBB	blood-brain barrier
CNS	central nervous system
Rh123	rhodamine123
MDCKII	Madin–Darby canine renal epithelial cell line
CSCs	cancer stem cells
Hh	Hedgehog
PARPs	polymerases
EGFR	epidermal growth factor receptor
TKI	tyrosine kinase inhibitor
COX-2	Cyclooxygenase-2
PG	prostaglandin
BMI1	Moloney murine leukemia virus integration site 1
PRC1	Polycomb repressive complex 1
HES1	hairy and enhancer of split-1
CXCR4	chemokine receptor type 4
CXCL12	chemokine ligand type 12
APC	Anaphase Promoting Complex
Ki-67-LI	Kiel 67 Labelling Index
SOX2	sex determining region Y-box 2
BLTs	bispecific ligand-targeted toxins
EGFuPA	epidermal growth factor and urokinase
FMS	Feline McDonough Sarcoma.
L-asp	l-asparaginase
FLT3	FMT-tyrosine kinase 3
STAT	signal transducers and activators of transcription
MAPK	mitogen-activated protein kinase
AKT	protein kinase B
PCR	polymerase chain reaction
ERK	extracellular signal-regulated kinase
NF-kB	B-cell nuclear factor kappa-light-chain-enhancer
IKK	IK kinase
NEMO	essential modulator-binding domain of NF-kB
HIF-1α	hypoxia inducible factor-1α
Bcl-2	B-cell leukemia/lymphoma 2
Bax	Bcl-2 associated X
EphA2	ephrin A2
cSRC	Rous sarcoma
AURKA	Aurora kinases A
AURKB	Aurora kinases B
siRNA	small interfering RNA
shRNA	short hairpin RNA
EMT	Epithelial-mesenchymal transition
HER	epidermal growth factor
Wnt	wingless-type MMTV integration site
CAR	chimeric antigen receptor
PDL1	programmed cell death 1 ligand 1
Kit	receptor tyrosine kinase
HSP	heat shock protein
CY	Cytochrome

## References

[1] Ingham J., Ruan J.L., Coelho M.A. (2025). Breaking barriers: We need a multidisciplinary approach to tackle cancer drug resistance. BJC Rep..

[2] Cassidy A., Frickel S., Albert M., Prainsack B. (2016). One Medicine? Advocating (Inter)disciplinarity at the Interfaces of Animal Health, Human Health, and the Environment. Investigating Interdisciplinary Collaboration: Theory and Practice Across Disciplines.

[3] MacEwen E.G. (1990). Spontaneous tumors in dogs and cats: Models for the study of cancer biology and treatment. Cancer Metastasis Rev..

[4] Oh J.H., Cho J.Y. (2023). Comparative oncology: Overcoming human cancer through companion animal studies. Exp. Mol. Med..

[5] De Biase D., Baldassarre V., Piegari G., Rosato G., Caputo V., Pompameo M., Sarnelli P., Russo V., D’Angelo D., Papparella S. (2023). Animal sentinels and cancer registries: State of the art and new perspectives. Ann. Res. Oncol..

[6] Cekanova M., Ratore K. (2014). Animal models and therapeutic molecular targets of cancer: Utility and limitations. Drug Des. Dev. Ther..

[7] Thamm D., Dow S. (2009). How companion animals contribute to the fight against cancer in humans. Vet. Ital..

[8] Gardner L.H., Fenger J.M., London C.A. (2016). Dogs as a Model for Cancer. Annu. Rev. Anim. Biosci..

[9] Page R., Baneux P., Vail D.M., DUDA L., Olson P., Anestidou L., Dybdal N., Golab G., Shelton V., Salgaller M. (2016). Conduct, Oversight, and Ethical Considerations of Clinical Trials in Companion Animals with Cancer: Report of a Workshop on Best Practice Recommendations. J. Vet. Intern. Med..

[10] Regan D., Garcia K., Thamm D. (2019). Clinical, Pathological, and Ethical Considerations for the Conduct of Clinical Trials in Dogs with Naturally Occurring Cancer: A Comparative Approach to Accelerate Translational Drug Development. ILAR J..

[11] Nance R.L., Sajib A.M., Smith B.F. (2022). Canine models of human cancer: Bridging the gap to improve precision medicine. Prog. Mol. Biol. Transl. Sci..

[12] Zandvliet M., Teske E. (2015). Mechanisms of Drug Resistance in Veterinary Oncology— A Review with an Emphasis on Canine Lymphoma. Vet. Sci..

[13] Klopfleisch R., Kohn B., Gruber A.D. (2016). Mechanisms of tumor resistance against chemotherapeutic agents in veterinary oncology. Vet. J..

[14] Tan L., Wang Y., Hu X., Du G., Tang X., Min L. (2023). Advances of Osteosarcoma Models for Drug Discovery and Precision Medicine. Biomolecules.

[15] Roulet A., Puel O., Gesta S., Lepage J.F., Drag M.D., Soll M.D., Alvinerie M., Pineau T. (2003). MDR1-deficient genotype in Collie dogs hypersensitive to the P-glycoprotein substrate ivermectin. Eur. J. Pharmacol..

[16] Shoieb A., Hahn K. (1997). Detection and significance of glutathione-S-transferase pi in osteogenic tumors of dogs. Int. J. Oncol..

[17] Mealey K.L., Bentjen S.A. (2003). Sequence and structural analysis of the presumed downstream promoter of the canine mdr1 gene. Vet. Comp. Oncol..

[18] Mealey K.L., Barhoumi R., Rogers K., Kochevar D.T. (1998). Doxorubicin induced expression of P-glycoprotein in a canine osteosarcoma cell line. Cancer Lett..

[19] Selvarajah G.T., Kirpensteijn J., van Wolferen M.E., Rao N.A.S., Fieten H., Mol J.A. (2009). Gene expression profiling of canine osteosarcoma reveals genes associated with short and long survival times. Mol. Cancer.

[20] Uozurmi K., Nakaichi M., Yamamoto Y., Une S., Taura Y. (2005). Development of multidrug resistance in a canine lymphoma cell line. Res. Vet. Sci..

[21] Zandvliet M., Teske E., Chapuis T., Fink-Gremmels J., Schrickx J.A. (2013). Masitinib reverses doxorubicin resistance in canine lymphoid cells by inhibiting the function of P-glycoprotein. J. Vet. Pharmacol. Therap..

[22] Zandvliet M., Teske E., Schrickx J.A. (2014). Multi-drug resistance in a canine lymphoid cell line due to increased P-glycoprotein expression, a potential model for drug-resistant canine lymphoma. Toxicol. In Vitro.

[23] Moore A.S., Leveille C.R., Reimann K.A., Shu H., Arias I.M. (1995). The Expression of P-Glycoprotein in Canine Lymphoma and Its Association with Multidrug Resistance. Cancer Investig..

[24] Bakos E., Evers R., Szakács G., Tusnády G.E., Welker E., Szabó K., de Haas M., van Deemter L., Borst P., Váradi A. (1998). Functional multidrug resistance protein (MRP1) lacking the N-terminal transmembrane domain. J. Biol. Chem..

[25] Zhang Y., Laterra J., Pomper M.G. (2009). Hedgehog Pathway Inhibitor HhAntag691 Is a Potent Inhibitor of ABCG2/BCRP and ABCB1/Pgp. Neoplasia.

[26] Parrish K.E., Cen L., Murray J., Calligaris D., Kizilbash S., Mittapalli R.K., Carlson B.L., Schroeder M.A., Sludden J., Boddy A.V. (2015). Efficacy of PARP Inhibitor Rucaparib in Orthotopic Glioblastoma Xenografts Is Limited by Ineffective Drug Penetration into the Central Nervous System. Mol. Cancer Ther..

[27] Salphati L., Alicke B., Heffron T.P., Shahidi-Latham S., Nishimura M., Cao T., Carano R.A., Cheong J., Greve J., Koeppen H. (2016). Brain Distribution and Efficacy of the Brain Penetrant PI3K Inhibitor GDC-0084 in Orthotopic Mouse Models of Human Glioblastoma. Drug Metab. Dispos..

[28] Norouzi M., Yathindranath V., Thliveris J.A., Kopec B.M., Siahaan T.J., Miller D.W. (2020). Doxorubicin-loaded iron oxide nanoparticles for glioblastoma therapy: A combinational approach for enhanced delivery of nanoparticles. Sci. Rep..

[29] Sane R., Agarwal S., Mittapalli R.K., Elmquist W.F. (2013). Saturable active efflux by p-glycoprotein and breast cancer resistance protein at the blood-brain barrier leads to nonlinear distribution of elacridar to the central nervous system. J. Pharmacol. Exp. Ther..

[30] Ballard P., Yates J.W., Yang Z., Kim D.W., Yang J.C., Cantarini M., Pickup K., Jordan A., Hickey M., Grist M. (2016). Preclinical Comparison of Osimertinib with Other EGFR-TKIs in EGFR-Mutant NSCLC Brain Metastases Models, and Early Evidence of Clinical Brain Metastases Activity. Clin. Cancer Res..

[31] Colclough N., Chen K., Johnström P., Strittmatter N., Yan Y., Wrigley G.L., Schou M., Goodwin R., Varnäs K., Adua S.J. (2021). Preclinical Comparison of the Blood-brain barrier Permeability of Osimertinib with Other EGFR TKIs. Clin. Cancer. Res..

[32] Hu Z., Zhou Z., Hu Y., Wu J., Li Y., Huang W. (2015). HZ08 reverse P-glycoprotein mediated multidrug resistance in vitro and in vivo. PLoS ONE.

[33] Krapf M.K., Gallus J., Wiese M. (2017). 4-Anilino-2-pyridylquinazolines and -pyrimidines as Highly Potent and Nontoxic Inhibitors of Breast Cancer Resistance Protein (ABCG2). J. Med. Chem..

[34] Hofman J., Sorf A., Vagiannis D., Sucha S., Kammerer S., Küpper J.H., Chen S., Guo L., Ceckova M., Staud F. (2019). Brivanib Exhibits Potential for Pharmacokinetic Drug-Drug Interactions and the Modulation of Multidrug Resistance through the Inhibition of Human ABCG2 Drug Efflux Transporter and CYP450 Biotransformation Enzymes. Mol. Pharm..

[35] Riganti C., Contino M., Guglielmo S., Perrone M.G., Salaroglio I.C., Milosevic V., Giampietro R., Leonetti F., Rolando B., Lazzarato L. (2018). Design, biological evaluation, and molecular modeling of tetrahydroisoquinoline derivatives: Discovery of a potent P-glycoprotein ligand overcoming multidrug resistance in cancer stem cells. J. Med. Chem..

[36] Guberović I., Marjanović M., Mioč M., Ester K., Martin-Kleiner I., Šumanovac Ramljak T., Mlinarić-Majerski K., Kralj M. (2018). Crown ethers reverse P-glycoprotein-mediated multidrug resistance in cancer cells. Sci. Rep..

[37] Ong S.M., Yamamoto H., Saeki K., Tanaka Y., Yoshitake R., Nishimura R., Nakagawa T. (2017). Anti-neoplastic effects of topoisomerase inhibitors in canine mammary carcinoma, melanoma, and osteosarcoma cell lines. Jpn. J. Vet. Res..

[38] Palko-Łabuz A., Błaszczyk M., Środa-Pomianek K., Wesołowska O. (2021). Isobavachalcone as an Active Membrane Perturbing Agent and Inhibitor of ABCB1 Multidrug Transporter. Molecules.

[39] Pang L.Y., Gatenby E.L., Kamida A., Whitelaw B.A., Hupp T.R., Argyle D.J. (2014). Global gene expression analysis of canine osteosarcoma stem cells reveals a novel role for COX-2 in tumour initiation. PLoS ONE.

[40] Shahi M.H., York D., Gandour-Edwards R., Withers S.S., Holt R., Rebhun R.B. (2015). BMI1 is expressed in canine osteosarcoma and contributes to cell growth and chemotherapy resistance. PLoS ONE.

[41] Dailey D.D., Anfinsen K.P., Pfaff L.E., Ehrhart E.J., Charles J.B., Bønsdorff T.B., Thamm D.H., Powers B.E., Jonasdottir T.J., Duval D.L. (2013). HES1, a target of Notch signaling, is elevated in canine osteosarcoma, but reduced in the most aggressive tumors. BMC Vet. Res..

[42] Al-Nadaf S., Peacott-Ricardos K.S., Dickinson P.J., Rebhun R.B., York D. (2022). Expression and therapeutic targeting of BMI1 in canine gliomas. Vet. Comp. Oncol..

[43] Gatti M., Solari A., Pattarozzi A., Campanella C., Thellung S., Maniscalco L., De Maria R., Würth R., Corsaro A., Bajetto A. (2018). In vitro and in vivo characterization of stem-like cells from canine osteosarcoma and assessment of drug sensitivity. Exp. Cell Res..

[44] Arnason T.G., MacDonald-Dickinson V., Gaunt M.C., Davies G.F., Lobanova L., Trost B., Gillespie Z.E., Waldner M., Baldwin P., Borrowman D. (2022). Activation of the Anaphase Promoting Complex Reverses Multiple Drug Resistant Cancer in a Canine Model of Multiple Drug Resistant Lymphoma. Cancers.

[45] Barbieri F., Thellung S., Ratto A., Carra E., Marini V., Fucile C., Bajetto A., Pattarozzi A., Würth R., Gatti M. (2015). In vitro and in vivo antiproliferative activity of metformin on stem-like cells isolated from spontaneous canine mammary carcinomas: Translational implications for human tumors. BMC Cancer.

[46] Liu W., Moulay M., Willenbrock S., Roolf C., Junghanss C., Ngenazahayo A., Nolte I., Escobar H.M. (2015). Comparative Characterization of Stem Cell Marker Expression, Metabolic Activity and Resistance to Doxorubicin in Adherent and Spheroid Cells Derived from the Canine Prostate Adenocarcinoma Cell Line CT1258. Anticancer. Res..

[47] Miranda I.C., Miller A.D. (2021). SOX2 Expression in Canine Neoplasia. Vet. Pathol..

[48] Schappa J.T., Frantz A.M., Gorden B.H., Dickerson E.B., Vallera D.A., Modiano J.F. (2013). Hemangiosarcoma and its cancer stem cell subpopulation are effectively killed by a toxin targeted through epidermal growth factor and urokinase receptors. Int. J. Cancer.

[49] Smallwood T.L., Small G.W., Suter S.E., Richards K.L. (2013). Expression of asparagine synthetase predicts *in. vitro.* response to L-asparaginase in canine lymphoid cell lines. Leuk. Lymphoma.

[50] Montaner-Angoiti E., Marín-García P.J., Llobat L. (2023). Epigenetic Alterations in Canine Malignant Lymphoma: Future and Clinical Outcomes. Animals.

[51] Suter S.E., Small G.W., Seiser E.L., Thomas R., Breen M., Richards K.L. (2011). FLT3 mutations in canine acute lymphocytic leukemia. BMC Cancer.

[52] Gaurnier-Hausser A., Patel R., Baldwin A.S., May M.J., Mason N.J. (2011). NEMO-binding domain peptide inhibits constitutive NF-κB activity and reduces tumor burden in a canine model of relapsed, refractory diffuse large B-cell lymphoma. Clin. Cancer Res..

[53] Gaurnier-Hausser A., Mason N.J. (2015). Assessment of canonical NF-κB activity in canine diffuse large B-cell lymphoma. Methods Mol. Biol..

[54] Schlein L.J., Thamm D.H. (2022). Review: NF-kB activation in canine cancer. Vet. Pathol..

[55] Li R., Wu H., Sun Y., Zhu J., Tang J., Kuang Y., Li G. (2021). A Novel Canine Mammary Cancer Cell Line: Preliminary Identification and Utilization for Drug Screening Studies. Front. Vet. Sci..

[56] Ou G., Jiang X., Gao A., Li X., Lin Z., Pei S. (2022). Celastrol Inhibits Canine Mammary Tumor Cells by Inducing Apoptosis via the Caspase Pathway. Front. Vet. Sci..

[57] O’Donoghue L.E., Ptitsyn A.A., Kamstock D.A., Siebert J., Thomas R.S., Duval D.L. (2010). Expression profiling in canine osteosarcoma: Identification of biomarkers and pathways associated with outcome. BMC Cancer.

[58] Harris E.D., Sharpe J.C., Strozen T., Abdi S., Kliewer M., Sanchez M.G., Hogan N.S., MacDonald-Dickinson V., Vizeacoumar F.J., Toosi B.M. (2024). The EphA2 Receptor Regulates Invasiveness and Drug Sensitivity in Canine and Human Osteosarcoma Cells. Cells.

[59] Cannon C.M., Pozniak J., Scott M.C., Ito D., Gorden B.H., Graef A.J., Modiano J.F. (2015). Canine osteosarcoma cells exhibit resistance to aurora kinase inhibitors. Vet. Comp. Oncol..

[60] Lund J.R., Paoloni M., Kurzman I., Padilla M., Argyle D.J. (2008). Inhibition of canine telomerase in vitro and in vivo using RNAi: Further development of a natural canine model for telomerase-based cancer therapies. Vet. J..

[61] Elshafae S.M., Kohart N.A., Altstadt L.A., Dirksen W.P., Rosol T.J. (2017). The Effect of a Histone Deacetylase Inhibitor (AR-42) on Canine Prostate Cancer Growth and Metastasis. Prostate.

[62] Timmermans-Sprang E.P.M., Mestemaker H.M., Steenlage R.R., Mol J.A. (2019). Dasatinib inhibition of cSRC prevents the migration and metastasis of canine mammary cancer cells with enhanced Wnt and HER signalling. Vet. Comp. Oncol..

[63] Terragni R., Casadei Gardini A., Sabattini S., Bettini G., Amadori D., Talamonti C., Vignoli M., Capelli L., Saunders J.H., Ricci M. (2014). EGFR, HER-2 and KRAS in Canine Gastric Epithelial Tumors: A Potential Human Model?. PLoS ONE.

[64] Gao Y., Packeiser E.M., Wendt S., Sekora A., Cavalleri J.M.V., Pratscher B., Alammar M., Hühns M., Brenig B., Junghanss C. (2024). Cross-Species Comparison of the Pan-RAF Inhibitor LY3009120′s Anti-Tumor Effects in Equine, Canine, and Human Malignant Melanoma Cell Lines. Genes.

[65] Cronise K.E., Hernandez B.G., Gustafson D.L., Duval D.L. (2019). Identifying the ErbB/MAPK Signaling Cascade as a Therapeutic Target in Canine Bladder Cancer. Mol. Pharmacol..

[66] Sakai O., Igase M., Mizuno T. (2020). Optimization of canine CD20 chimeric antigen receptor T cell manufacturing and in vitro cytotoxic activity against B-cell lymphoma. Vet. Comp. Oncol..

[67] Park J.S., Whiters S.S., Modiano J.F., Kent M.S., Chen M., Luna J.I., Culp W.T.N., Sparger E.E., Rebhun R.B., Monjazeb A.M. (2016). Canine cancer immunotherapy studies: Linking mouse and human. J. Immunother. Cancer.

[68] Lin T.Y., Bear M., Du Z., Foley K.P., Ying W., Barsoum J., London C. (2008). The novel HSP90 inhibitor STA-9090 exhibits activity against Kit-dependent and -independent malignant mast cell tumors. Exp. Hematol..

[69] Nance R.L., Cooper S.J., Starenki D., Wang X., Matz B., Lindley S., Smith A.N., Smith A.A., Bergman N., Sandey M. (2022). Transcriptomic Analysis of Canine Osteosarcoma from a Precision Medicine Perspective Reveals Limitations of Differential Gene Expression Studies. Genes.

[70] Nance R.L., Wang X., Sandey M., Matz B.M., Thomas A., Smith B.F. (2023). Single-Nuclei Multiome (ATAC + Gene Expression) Sequencing of a Primary Canine Osteosarcoma Elucidates Intra-Tumoral Heterogeneity and Characterizes the Tumor Microenvironment. Int. J. Mol. Sci..

[71] Court M.H. (2013). Canine cytochrome P450 (CYP) pharmacogenetics. Vet. Clin. N. Am. Small Anim. Pract..

[72] McFadyen M.C.E., Melvin W.T., Murray G.I. (2004). Cytochrome P450 enzymes: Novel options for cancer therapeutics. Mol. Cancer Ther..

[73] Chon E., Hendricks W., White M., Rodrigues L., Haworth D., Post G. (2024). Precision Medicine in Veterinary Science. Vet. Clin. N. Am. Small. Anim. Pract..

[74] Dujon A.M., Brown J.S., Destoumieux-Garzón D., Vittecoq M., Hamede R., Tasiemski A., Boutry J., Tissot S., Alix-Panabieres C., Pujol P. (2021). On the need for integrating cancer into the One Health perspective. Evol. Appl..

[75] Leone L. (2025). L’approccio One Health nella Legislazione Europea sugli Animali: Orientamenti e Prospettive. Eurojus.it. https://rivista.eurojus.it/wp-content/uploads/pdf/Leone-One-Health-eurojus.pdf.

[76] Coli F., Schebesta H. (2023). One Health in the EU: The Next Future?. Eur. Pap..

[77] La Torre G., Barbato D., Colamesta V., Lia L., Lombardi A.M., D Cacchio D., P Villari P., De Giusti M. (2018). Collaboration between human and veterinary medicine in Europe: A systematic review. Eur. J. Public Health.

[78] Alshammari A.H., Oshiro T., Ungkulpasvich U., Yamaguchi J., Morishita M., Khdair S.A., Hatakeyama H., Hirotsu T., di Luccio E. (2025). Advancing Veterinary Oncology: Next-Generation Diagnostics for Early Cancer Detection and Clinical Implementation. Animals.

[79] Stephens T. (2019). The Use of Chemotherapy to Prolong the Life of Dogs Suffering from Cancer: The Ethical Dilemma. Animals.

[80] Stoewen D.L., Coe J.B., MacMartin C., Stone E.A., Dewey C.E. (2019). Identification of Illness Uncertainty in Veterinary Oncology: Implications for Service. Front. Vet. Sci..

